# Noncoding RNA of Zika Virus Affects Interplay between Wnt-Signaling and Pro-Apoptotic Pathways in the Developing Brain Tissue

**DOI:** 10.3390/v15051062

**Published:** 2023-04-26

**Authors:** Andrii Slonchak, Harman Chaggar, Julio Aguado, Ernst Wolvetang, Alexander A. Khromykh

**Affiliations:** 1 School of Chemistry and Molecular Biosciences, The University of Queensland, Brisbane 4072, Australia; 2 Australian Infectious Diseases Research Centre, Global Virus Network Centre of Excellence, Brisbane 4072, Australia; 3 Australian Institute for Bioengineering and Nanotechnology, The University of Queensland, Brisbane 4072, Australia

**Keywords:** Zika virus, sfRNA, flavivirus, systems virology, transcriptomics, noncoding RNA, brain development, apoptosis, Wnt-signaling

## Abstract

Zika virus (ZIKV) has a unique ability among flaviviruses to cross the placental barrier and infect the fetal brain causing severe abnormalities of neurodevelopment known collectively as congenital Zika syndrome. In our recent study, we demonstrated that the viral noncoding RNA (subgenomic flaviviral RNA, sfRNA) of the Zika virus induces apoptosis of neural progenitors and is required for ZIKV pathogenesis in the developing brain. Herein, we expanded on our initial findings and identified biological processes and signaling pathways affected by the production of ZIKV sfRNA in the developing brain tissue. We employed 3D brain organoids generated from induced human pluripotent stem cells (ihPSC) as an ex vivo model of viral infection in the developing brain and utilized wild type (WT) ZIKV (producing sfRNA) and mutant ZIKV (deficient in the production of sfRNA). Global transcriptome profiling by RNA-Seq revealed that the production of sfRNA affects the expression of >1000 genes. We uncovered that in addition to the activation of pro-apoptotic pathways, organoids infected with sfRNA-producing WT, but not sfRNA-deficient mutant ZIKV, which exhibited a strong down-regulation of genes involved in signaling pathways that control neuron differentiation and brain development, indicating the requirement of sfRNA for the suppression of neurodevelopment associated with the ZIKV infection. Using gene set enrichment analysis and gene network reconstruction, we demonstrated that the effect of sfRNA on pathways that control brain development occurs via crosstalk between Wnt-signaling and proapoptotic pathways.

## 1. Introduction

Zika Virus (ZIKV) is an emerging pathogen that belongs to the genus *Flavivirus* in the family *Flaviviridae* [1]. It is a small, enveloped virus with a single-stranded RNA genome of positive polarity. ZIKV is a significant public health concern with a recent outbreak resulting in almost 1,000,000 infections [2]. In urban and suburban environments, ZIKV can circulate between humans and mosquitoes [3]. Common symptoms of ZIKV infection include papular rash, arthritis, myalgia, non-purulent conjunctivitis, headache, fever, retro-orbital pain, edema, and vomiting [4]. In addition, a temporal and geographical association between Guillain-Barre syndrome and ZIKV outbreaks in the Pacific and the Americans has been described, suggesting the infections may lead to neurological complications, which is a common disease outcome for flaviviruses [4]. Moreover, ZIKV is the only known flavivirus to have teratogenic effects in humans [5]. Mothers with proven antecedent acute ZIKV infections had high rates of giving birth to infants with microcephaly, visual impairment, and global neurodevelopmental delay [6,7]. In 2016, the World Health Organization announced ZIKV outbreaks as a health emergency of international concern, stressing the importance of further research on this virus. To date, no specific antiviral and vaccines have been approved for use against the ZIKV infection and the pathogenesis of ZIKV in the developing brain is not completely understood.

One of the characteristics of flaviviral infection is the intracellular accumulation of a virus-derived, non-coding RNA, referred to as subgenomic flavivirus RNA or sfRNA [8]. It is produced from the viral 3′UTRs due to the unique ability of flavivirus RNA to resist degradation by the host 5′-3′ exoribonuclease XRN1 [8,9]. The 3′UTRs of mosquito-borne flaviviruses commonly contain at least two highly structured RNA elements that can stall XRN1 progression, resulting in the production of at least two sfRNA species of slightly different lengths [10,11]. These RNA elements are usually referred to as XRN1-resistant RNA elements (xrRNAs). The production of sfRNAs has been shown in all flaviviruses tested to date [11,12,13], including phylogenetically divergent dual-host flaviviruses and insect-specific flaviviruses [11]. They were demonstrated to promote viral replication in arthropod and vertebrate hosts and facilitate the transmission of mosquito-borne flaviviruses (reviewed in [12,14]). ZIKV was shown to produce two sfRNA species of different lengths due to the presence of two XRN1-resistant elements in the 3′UTR [15,16]. In the insect host, the production of sfRNA was demonstrated to facilitate ZIKV replication and secretion into saliva by inhibiting the apoptosis of infected cells in mosquito tissues [16]. In vertebrate hosts, ZIKV sfRNA was found to facilitate viral replication and promote apoptosis and cytopathic effects [17,18], which is opposite to its inhibitory effect on apoptosis in mosquitoes. It was also shown to be required for viral dissemination through the placental barrier into the fetal brain [17]. ZIKV sfRNA was shown to execute its functions by suppressing interferon signaling [17,18] and promoting apoptosis, acting in cooperation with the viral protein NS5 [17].

In a recent study, we employed human brain organoids as a model of the ZIKV infection in the developing brain and found that ZIKV sfRNA also promotes viral replication in neural tissues and is required for viral neuropathic effect as well as for virus-induced deaths of neural progenitor cells (NPCs) accompanied by the activation of Caspase-3 [17]. Brain organoids are stem-cell-derived 3D tissues that self-assemble into the structures of the developing brain, utilizing the intrinsic ability of human pluripotent stem cells (hPSCs) to differentiate spontaneously into neural lineages [19]. They can be generated from embryonic (ehPSCs) or induced (ihPSCc) stem cells to resemble the entire brain (cerebral organoids) or be guided to become region-specific [19]. The advantage of organoids compared to cell monolayers is their ability to produce all relevant cell types and recapitulate the architecture of the developing brain. The human origin of organoids and their capacity to mount an innate immune response make them the most relevant accessible brain model for ZIKV research [20].

In this study, we employed ihPSCc-derived human brain organoids to further characterize the role of sfRNA in ZIKV pathogenesis in the developing brain. We performed transcriptome-wide gene expression profiling of organoids infected with WT ZIKV and the sfRNA-deficient mutant virus and identified genes that were differentially expressed compared to the mock. We then compared differentially expressed genes (DEGs) for each condition and identified those that were affected by the production of sfRNA. Using gene ontology enrichment analysis and network reconstruction we further confirmed our earlier findings regarding the ability of ZIKV sfRNA to promote apoptosis. In addition, we found that the production of sfRNA has a previously unknown inhibitory effect on signalling pathways that regulate brain development. We demonstrated that this effect likely results from the induction of pro-apoptotic signaling, which interconnects with pathways that control neuron differentiation and the development of the nervous system. Thus, we demonstrated that sfRNA is an important viral factor of ZIKV pathogenesis in the developing brain.

## 2. Materials and Methods

Generation of human brain organoids. Generations of human brain organoids have been described previously [21]. Human embryonic stem cell line GENEA022 (Genea Biocells, San Diego, CA, USA) was cultured on ECM Gel from Engelbreth-Holm-Swarm murine sarcoma (Sigma-Aldrich, St. Louis, MO, USA) in mTeSR medium (Stem Cell Technologies, Vancouver, BC, Canada). Brain organoids were generated using a protocol [19] in which patterned embryoid bodies were expanded for 4 days in N2 medium (DMEM/F12 (Gibco, Waltham, MA, USA) with 1% N-2 supplement (Gibco, USA), 2% B-27 supplement (Gibco, USA), 1% MEM Non-Essential Amino Acids (Gibco, USA), 100 U/mL Penicillin and 100 µg/mL Streptomycin (Gibco, USA), and 0.1% β-mercaptoethanol (Gibco, USA), with daily supplementation of 20 ng/mL bFGF (R&D, USA). Embryoid bodies were embedded in 15 µL of Matrigel (Stem Cell Technologies, Vancouver, BC, Canada) and media changed to a 1:1 mixture of Neurobasal medium (Invitrogen, Waltham, MA, USA) and DMEM/F12, containing 1:200 MEM-NEAA, 1:100 Glutamax (Gibco, Waltham, MA, USA), 1:100 N2 supplement, 1:50 B27 supplement, 1 s penicillin-streptomycin, 50 μM 2-mercaptoethanol, and 0.25% insulin solution (Sigma-Aldrich, St. Louis, MO, USA). Fresh media was replaced three times a week.

Viruses and infection. WT and sfRNA-deficient ZIKV were of the MR766 strain and have been described previously [16]. Brain organoids were utilized for a viral infection on day 15. A virus inoculum containing 10^5^ FFU of each virus was added to a single organoid-containing well of a 24-well plate and was incubated at 37 °C O/N. Each ZIKV-infected organoid was then washed three times with culture media and transferred to a single well of a 24-well plate containing 500 μL of ND medium.

Foci-forming immunoassay. Ten-fold serial dilutions of culture fluids were prepared in DMEM media, supplemented with 2% FBS and 25 µL of each dilution, and were used to infect 10^4^ Vero cells pre-seeded in 96 well plates. After 1 h of incubation at 37 °C, 180 µL overlay media (1:1 mixture of 2% carboxymethyl cellulose (Sigma-Aldrich, St. Louis, MO, USA) and 2× M199 medium supplemented with 5% FCS, 100 μg/mL streptomycin, 100 U/mL penicillin, and 2.2 g/L NaHCO_3_) was added to the wells. At 3 dpi, the overlay medium was removed and the cells were fixed with 100 µL/well of ice-cold fixation solution (80% acetone, 20% PBS) for 30 min at −20 °C. After the fixative solution was removed, the monolayer was fully dried and plates were blocked with 150 µL/well of ClearMilk blocking solution (Pierce, Waltham, MA, USA) for 60 min. The plates were then incubated for 1 h with 50 μL/well of 4G2 mouse monoclonal antibody specific toZIKV envelope protein in 1:100 dillution, followed by 1 h incubation with 50 μL/well of 1:1000 dilution of anti-mouse IRDye 800 CW secondary antibody (LI-COR Biosciences, Lincoln, NE, USA). All antibodies were diluted with Clear Milk blocking buffer (Pierce, Waltham, MA, USA) and incubations were performed at 37 °C. After each incubation with the antibodies, the plates were washed 5 times with phosphate-buffered saline containing 0.05% Tween 20 (PBST). The plates were scanned using an LICOR Odyssey CLx Imaging System with the following settings: channel = 800 and 700, quality= medium, focus= 3.0 mm, intensity = auto, resolution= 42 μm.

RNA isolation. Eight individual organoids were combined in 1 mL of TRIreagent (Sigma, USA) and homogenized for 5 min at 30 Hz using a Tissue Lyser II (Qiagen, Hilden, Germany). RNA was then isolated according to the TRIreagent manufacturer’s recommendations. The quantity and purity of the RNA were assessed using Nanodrop ND1000 (Thermo Fisher, Waltham, MA, USA).

Next-generation sequencing. RNA samples pooled from 8 brain organoids per sample, per condition, infected with WT or xrRNA2′ mutant ZIKV_MR766_ or mock-infected, were subjected to ribosomal RNA depletion using the Ribo-Zero Gold rRNA Removal Kit (Illumina, San Diego, CA, USA) followed by library preparation. Two pools of organoids were used in the experiment for each condition. Libraries were sequenced on an Illumina HiSeq4000 instrument generating single-end 150 bp reads. Image analysis was performed using the HiSeq Control Software (HCS) vHD 3.4.0.38 and Real-Time Analysis (RTA) v2.7.7 on the instrument computer. The Illumina bcl2fastq 2.20.0.422 pipeline was then used to produce the sequence data. Library preparation, sequencing, and data acquisition were performed by the University of Queensland Genomics Facility and the Australian Genomics Research Facility (AGRF). Quality control of sequencing data was performed using FastQC v.0.72. The data were then trimmed using TRIMMOMATIC v.0.36.4 and the following settings: ILLUMINACLIP: TruSeq3-PE:2:30:10 LEADING:32 TRAILING:32 SLIDINGWINDOW:4:20 MINLEN:25. Trimmed reads were mapped to the human genome assembly hg38 using HISAT2 v.2.1.0. A feature count was performed using HTSEQ v.0.9.1 in Union mode, with strand set to “Reverse” and end feature type set to “exon”. RNA-Seq data generated in this study are deposited in the Gene Expression Omnibus database (accession number GSE230197).

Differential gene expression analysis. Differential gene expression analysis was performed using edgeR v.3.24.0. Low abundance reads (<0.5 cpm) were removed from the data set and the data were normalized using the trimmed mean of the M-values method (TMM). Normalized data were used to build the quasi-likelihood negative binomial generalized log-linear model. The test for differential expression relative to a threshold (glmTreat) was then applied to the contrasts WT-Mock, Mut-Mock, and (WT-Mock)-(Mut-Mock) to identify the genes that had significantly different expressions between the conditions by at least two-fold. Genes were considered differentially expressed if the FDR-corrected *p*-values were <0.05. Gene expression data were plotted using ggplot2 v.3.3.2.

Gene ontology and pathway enrichment analyses. Gene ontology (GO) and KEGG pathway enrichment analyses were performed using the Database for Annotation, Visualization, and Integrated Discovery (DAVID) v6.8. Enrichment data were exported from DAVID, combined with gene expression values, and z-scores were calculated using the R package GOplot v.1.0.2. Heatmaps were generated using heatmap.2 function of R-package gplots v3.1.1.

Network reconstruction. Gene interaction networks were reconstructed using Cytoscape v3.8.0. Individual networks were generated from DEGs associated with GO categories related to brain development and apoptosis using a local search with the GeneMANIA Cytoscape Plug-in. Interaction types considered in the network were “pathways”, “genetic interactions”, and “physical interactions”. The networks were then merged using the “merge networks” function of Cytoscape, with the mode set to “Union” and the nod keys set to “gene name”. Betweenness centrality values were calculated using the “Analyze Network” function of Cytoscape and represent the numbers of the shortest paths that pass through each nod in the network. A table of gene names with logFC values was then merged with the table of nods where logFCs were displayed as nod coloring.

Quantitative RT-PCR. Total organoids RNA (500 ng) was used to produce cDNA with qScript cDNA SuperMix (Quantabio, USA) according to the manufacturer’s instructions. cDNA was diluted 1:10 and used as a template for qRT-PCR. PCR was performed in 20 µL of a reaction mix containing 1X SYBR Green PCR Master Mix (Applied Biosystems, Waltham, MA, USA), 10 pmoles of forward and reverse PCR primers, and 3 µL of diluted cDNA. Reactions were performed using the following cycling conditions: 95 °C for 5 min, 40 cycles of 95 °C for 5 s, and 60 °C for 20 s, followed by melting-curve analysis using QuantStudio 6 Flex Real Time PCR Instrument (Applied Biosystems, USA). Gene expression levels were normalized to *TBP*. Viral RNA levels were determined using the standard curve method by comparing the C_T_ values of the samples to the C_T_ values observed in the amplification of the serial dilutions (10^2^–10^8^ copies) of a PCR-amplified and purified ZIKV genomic fragment. For each experiment, RNA from 3 biological replicates was used and amplification of each cDNA sample was performed in technical triplicate. Negative controls were included for each set of primers. Primers for FOXG1, DLX5, and HIST1H1 are listed in Table S1. Primers for ISGs have been described previously [22].

Statistical analysis. Statistical analyses were performed using GraphPad Prism v.9.0 and R v4.1.2.

## 3. Results

### 3.1. Production of sfRNA Alters Transcriptome of ZIKV-Infected Human Brain Organoids

We recently showed that the production of sfRNA facilitates ZIKV replication in human brain organoids and is required for the viral neuropathic effect. Organoids infected with WT ZIKV exhibited signs of pathology starting from 6 days post-infection (dpi) and disintegrated by 15 dpi, while organoids infected with sfRNA-deficient ZIKV mutants survived the course of infection. We also found that organoids infected with the sfRNA-producing WT virus had stronger activation of Caspase-3 compared to those infected with the sfRNA-deficient mutant virus and demonstrated that the production of sfRNA induced the apoptosis of neural progenitor cells [17].

**Figure 1:**
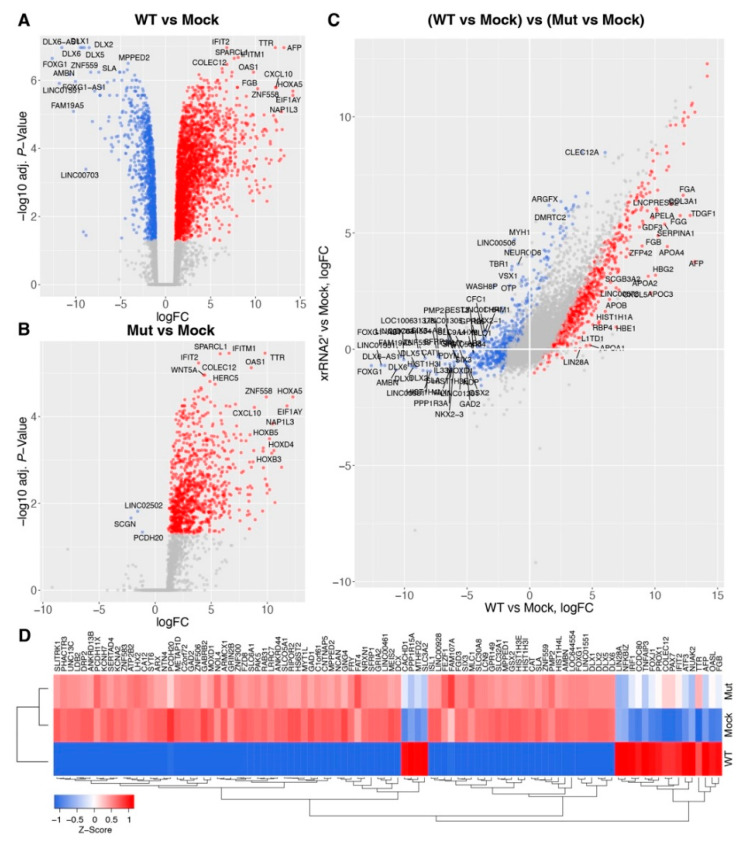
Differential host gene expression in iPSC-derived human brain organoids infected with WT and sfRNA-deficient ZIKV. Human brain organoids at DIV15 were infected with WT or sfRNA-deficient ZIKV mutant at a dose of 10^5^ FFU. Total RNA was isolated at 3 dpi and analyzed by RNA-Seq. (**A**) Volcano plot showing differentially expressed genes in human brain organoids infected with WT ZIKV. (**B**) Volcano plot showing differentially expressed genes in human brain organoids infected with sfRNA-deficient ZIKV mutant. In (**A**,**B**) genes with significantly different expression levels compared to the mock (FDR-corrected *p*-value < 0.05 and logFC > 1 or <−1) are shown in the color red for upregulated genes and blue for downregulated genes. The top most significant differentially expressed genes (DEGs) are labeled. (**C**) Comparison of gene expression levels between human brain organoids infected with WT and sfRNA-deficient (Mut) ZIKV. Genes with significantly different expressions (FDR-corrected *p*-value < 0.05 and logFC > 1 or <−1) are shown in the color red for genes with higher expression in WT infection compared to Mut virus infection and blue for genes with lower expression in WT infection compared to Mut virus infection. (**D**) Heat map representation of the expression levels of the top 100 most significant genes identified in (**C**).

**Figure 2:**
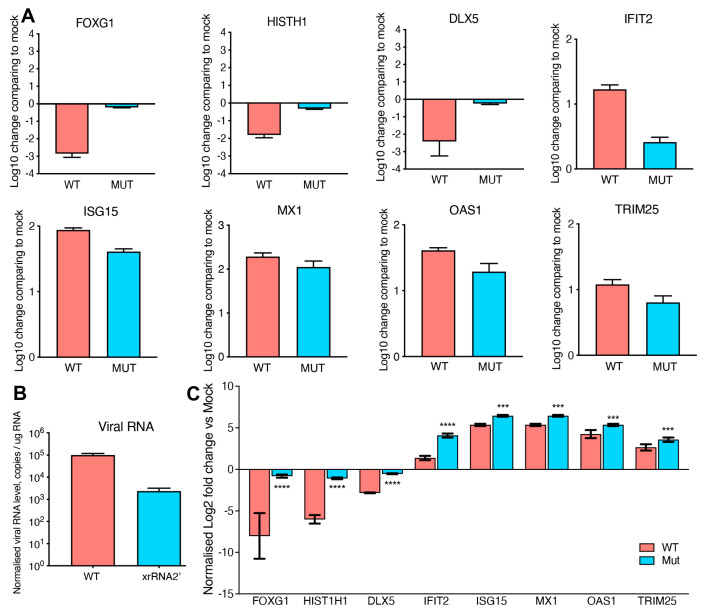
qRT-PCR validation of differentially expressed host genes identified by global transcriptome profiling. RNA isolated from human brain organoids infected with WT or sfRNA-deficient mutant viruses was subjected to cDNA synthesis and qRT-PCR. RNA from mock-infected organoids was used as a control. (**A**) Expression of the genes involved in brain development and ISGs determined using the ΔΔC_T_ method relative to the mock, with normalization to TBP mRNA level. (**B**) Viral RNA levels in infected organoids determined as copy numbers per ug of input RNA using the standard curve method. (**C**) Expression of host genes involved in brain development and ISGs normalized to viral RNA levels. The values are the means of three replicates ± SD. Statistical analysis was performed using Student’s *t*-test: **** *p* < 0.0001, *** *p* < 0.001.

To further dissect how the production of sfRNA affects developing brain tissues during the ZIKV infection we infected human brain organoids at day-in-vitro 15 (DIV15) with WT ZIKV and sfRNA-deficient mutant viruses and isolated RNA for transcriptome profiling at 3 dpi. This time point was selected because we previously determined that at 3 dpi, viral replication in brain organoids was at its highest level, while the morphology and tissue structure of organoids remained intact [17]. We employed a previously designed xrRNA2′ viral mutant that contains mutations in the XRN1-resistant structure xrRNA2 and produces neither sfRNA2 nor sfRNA1 in vertebrate cells [17]. RNA-Seq analysis demonstrated that infection with WT ZIKV led to the significant (FDR-corrected *p*-value < 0.05) up-regulation of 2334 genes and down-regulation of 1109 genes (Figure 1A and Table S2), while infection with sfRNA-deficient mutant virus significantly increased the expression of 971 genes and decreased the expression of only 3 genes (Figure 1B and Table S3). Consistent with previous observations [17,18], WT ZIKV induced the expression of interferon-stimulated genes (ISGs) (OAS1, IFIT2, IFITM1) and pro-inflammatory cytokines, e.g., CXCL10 (Figure 1A). Infection with the sfRNA-deficient ZIKV mutant also induced the expression of these genes, although to a lesser extent (Figure 2B). Notably, the down-regulated genes, that were specific to infection with the sfRNA-producing WT virus, included regulators of neuron differentiation, such as DLX6, DLX5, and FOXG1 (Figure 1A), which indicates that the production of sfRNA may contribute to the dysregulation of brain development in ZIKV infections.

Statistical comparisons of differentially expressed genes between brain organoids infected with WT ZIKV and sfRNA-deficient mutant ZIKV demonstrated that, in total, 1896 genes exhibited significantly different responses to infection with WT compared to the mutant virus (Figure 1C and Table S4). Amongst the genes with the most significant difference in expression between the WT and mutant virus infections were antiviral genes (e.g., IRF1, IFIT2, OASL) that exhibited a higher expression in the WT virus infection (Figure 2D). In addition, we found that other than the regulators of brain development, genes exclusively down-regulated in response to being infected with WT-virus included histones (e.g., HIST1H4L, HIST1H3E) and long noncoding RNAs (LINC00506, LINC01164, LINC00461, etc).

To validate the results of transcriptome profiling, we repeated the organoid infection and performed qRT-PCR for the mRNAs of several genes that exhibited significant differences in expression between organoids infected with WT and sfRNA-deficient ZIKV. We selected DLX5, HIST1H1, and FOXG1 as the representatives of downregulated genes and IFIT2, ISG15, MX1, OAS1, and TRIM25 as representatives of upregulated genes. The results of qRT-PCR were consistent with RNA-Seq data, showing significantly stronger down-regulation of FOXG1, HIST1H1, and DLX5 regarding WT virus infection compared to the mutant virus infection (Figure 2A). The expression of ISGs was also confirmed to be higher in WT ZIKV-infected organoids (Figure 2A). This finding was rather unexpected, as ZIKV sfRNA was previously shown by us and others to inhibit interferon (IFN) signaling and ISG expression in infected cells [17,18]. This discrepancy can be explained by the difference between the infections in the brain organoids used in this study and the infections in the cell monolayers utilized previously. As we recently showed, in brain organoids, ZIKV infects only a small proportion of cells [17], whereas, in a cell monolayer culture infected at a high multiplicity of infection, all cells are infected. As per the results, all cells grown in the monolayer culture should contain sfRNA and exhibit the same phenotype in regard to the antiviral response. In contrast, organoids have a large number of uninfected cells that do not contain sfRNA and also respond to IFNs produced by infected cells. As IFN production itself is not affected by sfRNA, we should expect that organoids infected with the WT virus, which exhibits higher replication efficiency, would produce more IFN, triggering a stronger antiviral response in uninfected bystander cells. In this case, the expression of ISGs should be proportional to the amount of viral RNA present in the organoids. To test this hypothesis, we determined the viral copy number in organoids infected with WT and sfRNA-deficient ZIKV (Figure 2B) and normalized gene expression to the viral RNA (Figure 2C). We found that the expression of ISGs per copy of viral RNA was significantly higher in organoids infected with the mutant virus compared to those with the WT infection, which is consistent with previous findings on the inhibitory effect of sfRNA on IFN signaling. Notably, the difference in the expression of FOXG1, HIST1H1, and DLX5 between organoids infected with WT and sfRNA-deficient viruses was still preserved after normalization (Figure 2C), which indicates that the unique effect of sfRNA on the suppression of these genes in infected cells cannot be attributed to the higher virus replication efficiency.

In summary, we demonstrated that the production of sfRNA by ZIKV results in the specific down-regulation of multiple genes in the developing brain tissue. Some of these genes are known to be involved in the regulation of brain development [23].

### 3.2. Production of sfRNA in ZIKV-Infected Human Brain Organoids Affects Activity of Multiple Pathways Related to Brain Development

To identify molecular processes and signaling pathways affected by the production of sfRNA in ZIKV-infected human brain organoids, the list of the most significantly affected genes (FDR-corrected *p*-value < 0.01 in Table S4) was subjected to gene ontology (GO) and KEGG pathway enrichment analyses. The information about the association between individual DEGs and enriched GO or KEGG categories was then combined with information about their logFC in WT vs mutant virus comparison to calculate z-scores that show how significantly each pathway or process was affected and in which direction the cumulative expression of the associated genes was shifted.

**Figure 3:**
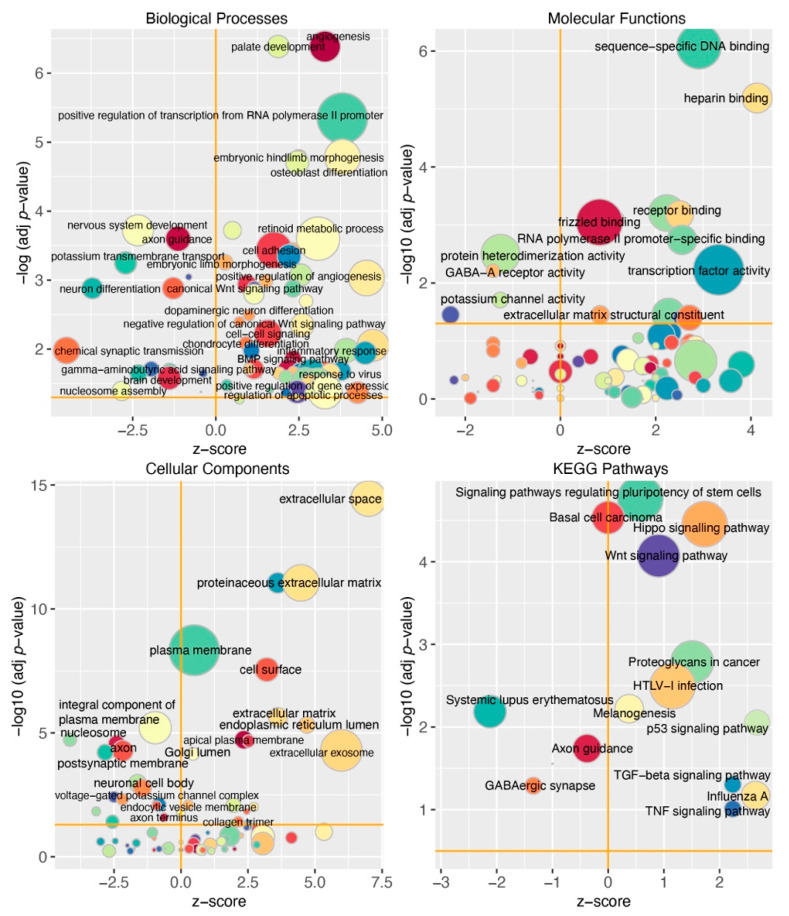
Functional classification of differentially expressed host genes that responded differently to infection with WT compared to sfRNA-deficient ZIKV in iPSC-derived human brain organoids. Size of bubbles on the plots is proportional to the number of associated genes; significantly enriched categories are labeled.

This analysis demonstrated that the production of sfRNA led to the inhibition of biological processes “nervous system development”, “neuron differentiation”, “axon guidance”, “brain development”, and “activation of Wnt-signaling”, which counteracts neuron differentiation (Figure 3). Biological processes (“regulation of apoptotic processes”, “inflammatory response”, and “response to virus”) exhibited higher activation in organoids infected with sfRNA-producing WT ZIKV compared to those infected with the sfRNA-deficient mutant (Figure 3). In molecular functions ontology, the enriched categories were related to DNA binding and transcription factor activity, and frizzled binding (Figure 3). As frizzled is a family of G protein-coupled receptors that mediate Wnt signaling [23], this further supports the conclusion regarding the effect of ZIKV sfRNA production on this pathway. The enrichment in cellular components ontology demonstrated that the production of sfRNA was associated with decreased expression of nucleosome components (Figure 3), which is consistent with reduced nucleosome assembly identified by biological process analysis. In addition, sfRNA-producing ZIKV was found to reduce the expression of genes that encode neuronal proteins, as indicated by negative z-scores observed for the categories “axon”, “postsynaptic membrane”, and “neuronal cell body”. This provides additional evidence for the inhibitory effect of sfRNA on neuron differentiation and brain development.

KEGG pathway enrichment analysis (Figure 3) demonstrated that the production of sfRNA was associated with increased activity of the Wnt pathway, Hippo signaling, inflammation (TNF-signaling), and p53-induced apoptosis, while the axon guidance pathway was suppressed by the sfRNA-producing virus. These results are generally consistent with GO enrichment analysis and provide additional evidence for the inhibition of brain development and activation of apoptosis resulting from sfRNA production. In addition, they identify the effect of sfRNA on Hippo signaling; however, the role of this pathway in the mammalian nervous system is not entirely clear, with different evidence suggesting its role in neuron differentiation as well as neuroinflammation and neuron apoptosis.

In summary, gene ontology and KEGG pathway enrichment analyses confirmed the previously identified pro-apoptotic function of ZIKV sfRNA in the developing neural tissue. In addition, it showed that the production of sfRNA by ZIKV is required for the inhibition of brain development and neuron differentiation.

### 3.3. ZIKV sfRNA Affects Brain Development via a Link between Pro-Apoptotic Signaling and Wnt Pathway

**Figure 4:**
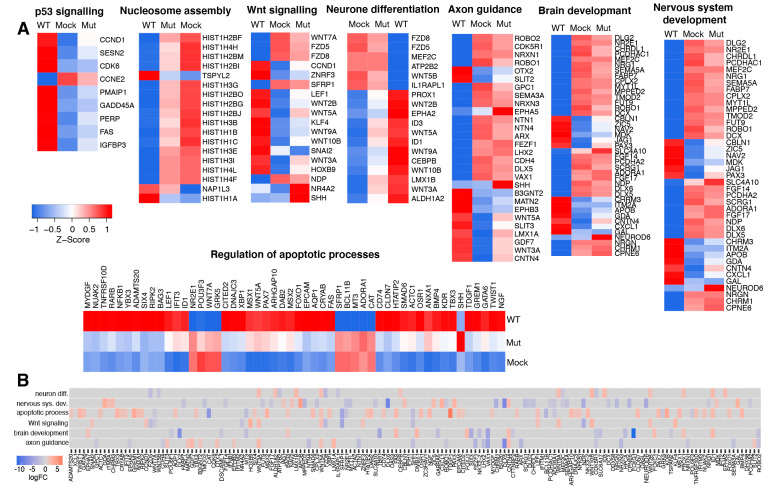
Expression of the host genes associated with brain development and antiviral response in iPSC-derived human brain organoids infected with WT and sfRNA-deficient ZIKV. (**A**) Expression of host genes associated with biological processes and signaling pathways differentially affected by WT and sfRNA-deficient ZIKV. (**B**) Heat map showing association with multiple biological processes of individual differentially expressed host genes.

To obtain an understanding of why the production of sfRNA results in the suppression of brain development, we looked at the associations between individual DEGs and the most affected biological processes and signaling pathways. We found that GO categories related to neural development had many shared genes and seemed in part synonymous (Figure 4A,B). We also noticed that these categories shared several genes with pathways related to apoptosis (e.g., WNT7A, WNT5A, SFRP1, Figure 4A,B). Therefore, we hypothesized that affected pathways may interconnect and, in some way, affect each other.

**Figure 5:**
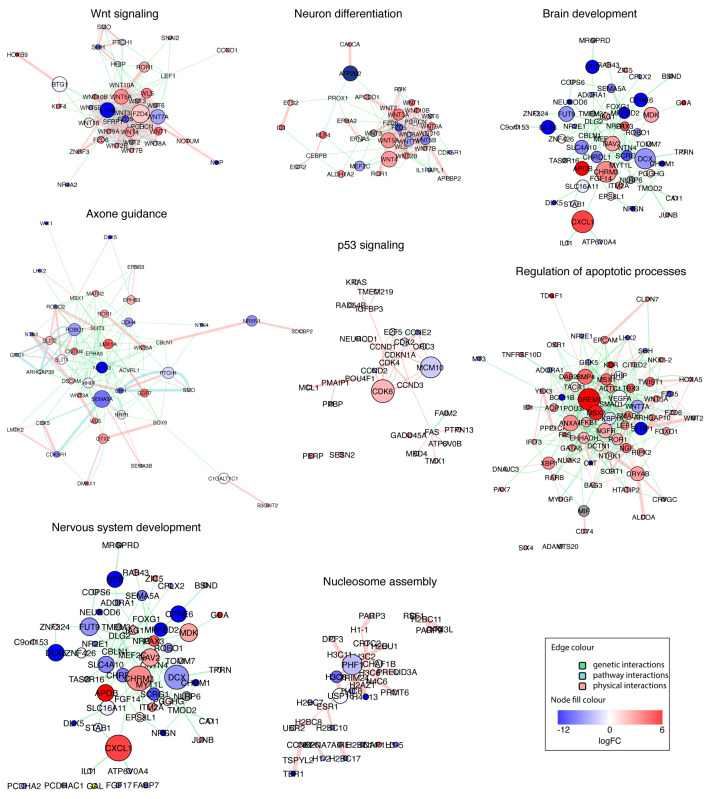
Networks of interactions between the genes affected by the production of sfRNA in ZIKV infection of human brain organoids. Genes associated with each enriched functional category shown in Figure 5A were used to reconstruct the networks of genetic, physical, and pathway interactions. Nod sizes indicate betweenness centrality, the logFC values are for ((WT−Mock) − (Mut−Mock)) contrast.

**Figure 6:**
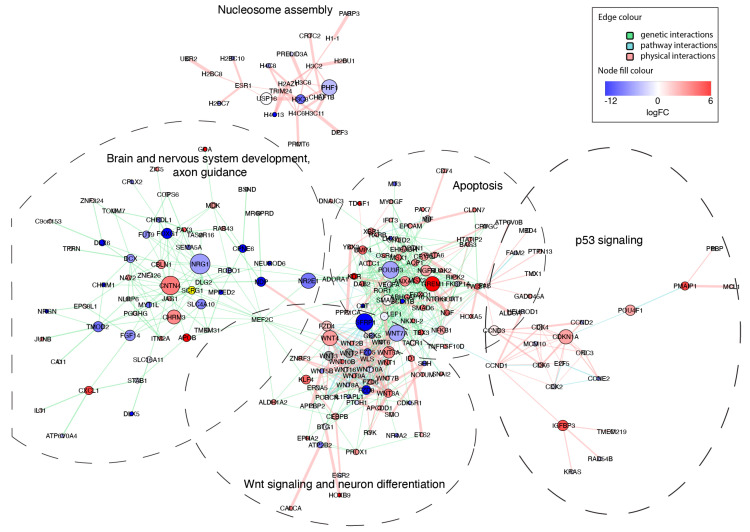
Interconnection between sfRNA-affected biological processes in ZIKV-infected human brain organoids. Individual networks were reconstructed from sfRNA-affected genes related to the most enriched pathways and GO BP terms: Wnt signaling pathway, nervous system development, neuron differentiation, axon guidance, brain development, regulation of apoptotic processes, p53 signaling pathway, and nucleosome assembly. Networks were then merged using the union method and gene names as query keys. The resulting network was analyzed to determine betweenness centrality values for the nods (visualized as nod size). The logFC values are for ((WT−Mock) − (Mut−Mock)) comparison.

To test this hypothesis, we reconstructed gene interaction networks for each biological pathway and GO category shown in Figure 4A (Figure 5), merged them, and explored the topology of the resulting network. The merged network consisted of five clusters, four of which were connected, which indicates cross-talk between all of the affected processes, except nucleosome assembly (Figure 6). Network analysis also demonstrated that networks reconstructed from genes associated with brain development and nervous system development were virtually identical (Figure 6), which indicates that these GO terms are highly synonymous. In the merged network, they formed a common cluster with axon guidance genes. Another major cluster in the resulting network consisted of combined genes related to Wnt signaling and neuron differentiation, which indicates that these processes are tightly interconnected (Figure 6). As expected, this cluster was connected to the “brain and nervous system developments, axon guidance” subnetwork (Figure 6). In addition, it appeared to be highly connected to the subnetwork reconstructed from apoptosis-related genes (Figure 6). Wnt signaling/neuron differentiation and apoptosis subnetworks contained multiple shared nods, including WNT7A, SFRP1, LEF1, and others (Figure 6). The connections between different functional categories of genes affected by the production of sfRNA in ZIKV-infected human brain organoids identified by network analysis suggest that the production of sfRNA likely affects brain development indirectly, due to connections between apoptosis pathways and Wnt-signaling, which in turn regulates neuron differentiation.

Therefore, we propose the following model to explain the inhibitory effect of sfRNA production on brain development. In ZIKV-infected neural progenitors, sfRNA triggers apoptosis. The activation of pro-apoptotic pathways also activates Wnt-signaling. The Wnt signaling pathway inhibits neuron differentiation, which leads to disruptions in brain development.

## 4. Discussion

The distinctive feature of ZIKV pathogenesis is the ability of the virus to pass through the placental barrier and establish infection in the fetal brain, causing neurodevelopment abnormalities, such as microcephaly [24]. To date, the molecular mechanisms responsible for this effect of the Zika infection are not fully understood. Our recent results demonstrated that sfRNA acts as one of the viral factors of ZIKV pathogenesis in the developing brain [17]. Production of sfRNA was also shown to be a requirement for trans-placental dissemination of the virus and infection of the fetal brain in the mouse pregnancy model [17,25]. In addition, in human brain organoids and cultured neural progenitors, WT virus-producing sfRNA induced the apoptosis of infected cells, while viruses deficient in sfRNA production were much less potent in inducing apoptosis and were unable to cause the death of the infected brain organoids [17].

Herein, we used the RNA-seq of ZIKV-infected human brain organoids to identify the entire spectrum of biological processes and signaling pathways affected by ZIKV sfRNA. Consistent with previous studies [26,27,28], we found that infection with WT ZIKV inhibits the expression of multiple genes involved in brain development, neuron differentiation, and nucleosome assembly, and it activates the expression of genes involved in the antiviral response, apoptosis, and inflammation. However, down-regulation of the genes involved in brain development and apoptosis in organoids infected with the sfRNA-deficient ZIKV mutant was not observed (Figure 1 and Figure 3). Notably, the activation of antiviral and pro-inflammatory genes was evident in organoids infected with the mutant virus, as well as in wild type infection, although it was less profound due to the lower viral loads. The comparable activation of antiviral genes by both viruses indicates that differences in replication between WT and mutant ZIKV only cause subtle quantitative differences in gene expression and are not responsible for the virtually non-existing effect of the sfRNA-deficient ZIKV infection on the expression of genes involved in brain development (Figure 1). Among the genes highly suppressed by WT, but not by sfRNA-deficient ZIKV, were FOXG1 and LHX2 (Figure 1A,B). FOXG1 is a transcription factor that has been previously linked to a variety of congenital brain disorders, including postnatal microcephaly [29]. LHX2 is another neural transcription factor that regulates neural differentiation by suppressing the Wnt signaling [30]. Decreased expression of these genes was previously identified as a risk factor for the development of ZIKV-induced microcephaly in a human discordant twins study [31]. In addition, ZIKV infection was shown to inhibit the expression of FOXG1 in primary human fetal neural progenitors [32]. Here, we demonstrate that this activity of ZIKV requires sfRNA.

One of the pathways highly affected by the production of ZIKV sfRNA during neuro-infection was Wnt-signaling. This pathway is generally involved in the regulation of development, and in neural development it controls the differentiation of neural progenitor cells [23]. Wnt signaling is initiated by the binding of secreted Wnt ligands to plasma membrane receptors of the Frizzled family and co-receptor of the low-density lipoprotein receptor-related protein family. The resulting receptor-ligand complex recruits Dishevelled protein (DVL), which in turn recruits the Axin and glycogen synthase kinase 3β (GSK3β) complex that are the components of the β-catenin destruction complex. This causes dissociation of the β-catenin destruction complex and leads to the stabilization and accumulation of β-catenin, which then translocates to the nucleus, forms a complex with TCF/LEF transcription factors, and regulates the expression of the target genes [33]. Dysregulation of the Wnt pathway during infection was previously proposed as a potential cause of ZIKV-associated microcephaly [34]. Brain development relies on the proliferation and differentiation of neural progenitor cells (NPC). NPCs can divide symmetrically, producing two NPCs or asymmetrically producing one NPC and one neuron. The path taken by the NPCs is controlled by the Wnt pathway, which inhibits differentiation and favors symmetric division. Wnt is in turn controlled by a transcription factor FOXG1, which inhibits Wnt pathway activity and promotes differentiation. According to our data, the production of sfRNA is associated with the downregulation of FOXG1 and up-regulation of multiple genes involved in the Wnt pathway (Figure 1 and Figure 4). Therefore, it should inhibit the asymmetric division of NPCs, thus preventing the generation of mature neurons and the formation of the brain.

Collectively, our data demonstrate that the production of ZIKV sfRNA is required for the inhibition of brain development via the activation of Wnt-signaling. However, it is unclear why sfRNA targets pathways involved in brain development if their suppression is unlikely to be beneficial for virus replication/survival. Therefore, we hypothesized that the effect of sfRNA on Wnt signaling can be indirect and result from crosstalk between Wnt-signaling and another pathway, which is the primary target of sfRNA relevant to virus-host interactions. This hypothesis was supported by gene interaction network analysis. It demonstrated that Wnt-signaling tightly interconnects with a network of pro-apoptotic genes activated by sfRNA (Figure 6). We recently demonstrated the requirement of sfRNA for the induction of apoptosis [17], and here we demonstrate that the production of sfRNA is associated with the activation of 8 genes that belong to p53 signaling and 42 genes related to other pro-apoptotic pathways, including Fas and TNF signaling (Figure 4 and Figure 5). Notably, some of the apoptosis-related genes (e.g., SFRP1, GRK5, WNT5A, WNT7A, and LEF1) were also components of the Wnt-signaling pathway (Figure 4B and Figure 6). Interconnection between Wnt-signaling and apoptosis was reported previously [35,36]. In particular, Wnt7a was shown to inhibit apoptosis [37,38]. Herein, we observed the inhibitory effect of ZIKV sfRNA on the expression of Wnt7a, which is consistent with the activation of apoptosis by sfRNA. In contrast, Wnt5a induces apoptosis [39] and it was up-regulated in brain organoids infected with WT, but not sfRNA-deficient ZIKV. Therefore, we propose that apoptosis is likely to be the primary target of the sfRNA-dependent modulation of Wnt-signaling, while brain development is affected indirectly due to the shared role of Wnt-signaling in both processes.

Another category of genes whose expression was affected by the production of ZIKV sfRNA was related to nucleosome assembly and was primarily represented by a different member of the histone 1H family. These genes were significantly more down-regulated by WT ZIKV compared to the sfRNA-deficient virus, except for HIST1H1A, which was up-regulated. In addition, the gene encoding for nucleosome assembly protein TSPYL2 was up-regulated only by the sfRNA-producing virus. The inhibitory effect of ZIKV infection on the expression of histones was shown previously [32]; however, the functional significance of this effect for viral infection is currently unclear. The H1 family is currently the most understudied group of histones, and their biological functions are not fully understood. In addition, our network analysis did not identify any connections between this group of genes and other pathways affected by sfRNA. Therefore, it is unclear why sfRNA causes suppression of the expression of the H1 family of histones. TSPYL2 is known as an inhibitor of cell-cycle progression [40] and activation of its expression may be related to cell-cycle arrest associated with ZIKV infection [41]. However, further research is required to understand the effect of the ZIKV infection on chromatin and the role of sfRNA in the modulation of nucleosome assembly.

In summary, our study for the first time identifies the effects of ZIKV sfRNA production on gene expression in the developing brain tissue, using human brain organoids as an ex vivo model of infection. It reveals the requirement of sfRNA for the inhibitory effect of ZIKV infection on neural development and identifies Wnt-signaling as the sfRNA-affected pathway responsible for it. Our comprehensive pathway enrichment and network reconstruction analysis establishes the link between the Wnt-pathway and pro-apoptotic activity of sfRNA and suggests that the inhibition of brain development by ZIKV sfRNA is an indirect effect of its function on the activation of apoptosis.

## Data Availability

All data is available within the manuscript and Supplementary Materials.

## Supplementary Materials

The following supporting information can be downloaded at: https://www.mdpi.com/article/10.3390/v15051062/s1, Table S1: sequences of the oligonucleotides used in the study; Table S2: differentially expressed genes in human brain organoids infected with WT ZIKV; Table S3: Differentially expressed genes in human brain organoids infected with sfRNA-deficient ZIKV mutant; Table S4: brain organoid genes with expression significantly affected by the production of sfRNA: ((WT-Mock)-(Mut-Mock)) contrast.
